# Resistant Polymorphic Ventricular Tachycardia in a Patient Taking Raspberry Ketones Weight Loss Supplement

**DOI:** 10.7759/cureus.33089

**Published:** 2022-12-29

**Authors:** Saad Ali Ansari, Femina Patel, Diana Ashouri, Jasninder Singh S Dhaliwal, Aditya Desai

**Affiliations:** 1 Internal Medicine, University of California Riverside School of Medicine, Riverside, USA

**Keywords:** transvenous pacing, obesity, raspberry ketones, weight loss supplements, polymorphic ventricular tachycardia

## Abstract

A 32-year-old female with no cardiac risk factors was admitted for treatment of a perianal abscess. During her hospital stay, she had a pulseless electrical activity arrest with a return of spontaneous circulation after one round of cardiopulmonary resuscitation (CPR). After transfer to the Intensive Care Unit (ICU), the patient had polymorphic ventricular tachycardia (PVT) requiring defibrillation shock. Her PVT was resistant to medical interventions. She was shocked a total of 33 times before her arrhythmia was terminated by passing a temporary transvenous pacemaker with overdrive pacing. After an extensive review of her history and presentation, no clear cause of her resistant arrhythmia was identified, however, she was found to have recently started taking over-the-counter weight loss supplements containing raspberry ketones which is a potentially cardiotoxic ingredient.

## Introduction

Obesity has become a major health problem worldwide and in the absence of an effective universal medical approach to tackle this epidemic, many patients use over-the-counter (OTC) weight loss supplements to help them lose weight. Numerous products are available that promote weight loss and appetite suppression and are sold as fat burners. These supplements are often sold without any regulatory process or having undergone any Food and Drug Administration (FDA) approved clinical trial showing safety and efficacy. As a result, an increasing number of toxicity cases caused by supplements are reported [1]. In this report, we are describing a case of a life-threatening polymorphic ventricular tachycardia (PVT) in a young female who had recently started taking an OTC fat burner containing raspberry ketones as an ingredient. The patient was admitted for treatment of a rectal abscess without any cardiac complaints. During hospitalization, she experienced resistant PVT that responded only to a temporary transvenous pacemaker with overdrive pacing. The increasing rate of reported toxicity of fat-burning supplements warrants attention by FDA to impose more regulation and oversight for this industry.

## Case presentation

**Figure 1:**
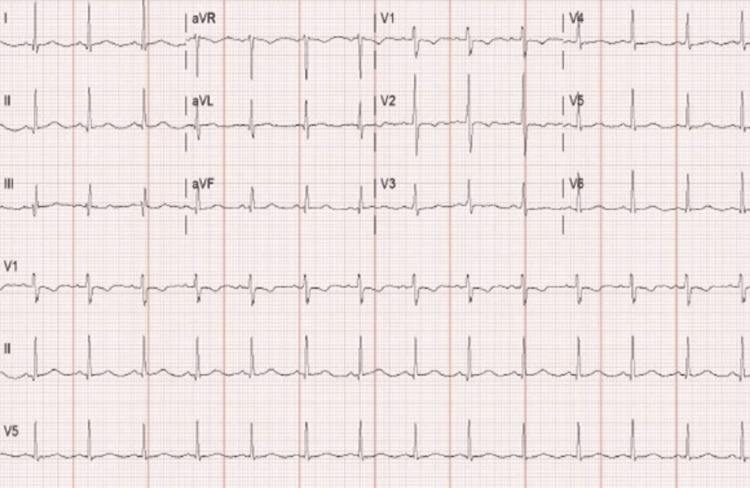
Baseline EKG. EKG: electrocardiogram

The patient was a 32-year-old obese female with no known past medical history who presented with fever and perianal pain for five days. She was found to have a perirectal abscess and underwent incision and drainage. A baseline laboratory work-up showed leukocytosis with a white blood cell count of 19.7 k/uL (4-11 k/uL). Serum electrolytes, kidney function, and liver function were normal. The baseline electrocardiogram (EKG) of the patient showed a heart rate of 83 with a QT of 393 and a borderline normal QTc of 462 (Figure 1). She was working with physical therapy on the floor when she became unresponsive and had a pulseless electrical activity (PEA) arrest. Code blue was called with the return of spontaneous circulation (ROSC) achieved after one round of cardiopulmonary resuscitation (CPR), and the patient was transferred to the Intensive Care Unit (ICU).

**Figure 2:**
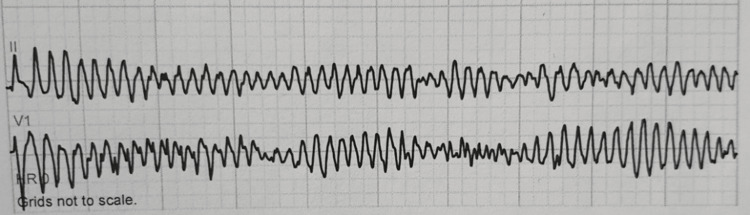
EKG showing polymorphic ventricular tachycardia. EKG: electrocardiogram

After transfer to the ICU, the patient had PVT requiring defibrillation shock (Figure 2). She briefly converted to sinus rhythm but the PVT recurred - over 2 hours she received 33 defibrillation shocks in addition to magnesium, calcium chloride, amiodarone, and lidocaine per Advanced Cardiac Life Support (ACLS) protocol. Amiodarone and lidocaine drips were initiated but on-call Cardiology recommended the discontinuation of amiodarone. When Cardiology arrived at the bedside, an emergent transvenous pacemaker was placed with overdrive pacing, which successfully terminated the arrhythmia. The patient’s baseline EKG was normal. Post arrhythmia, no QTc prolongation or shortening was identified on multiple EKGs and telemetry monitoring did not show any sinus pauses. Electrolytes were within normal limits. The urine drug screen was negative. Post-arrest echocardiogram showed a left ventricular ejection fraction of 20% with global hypokinesis possibly secondary to myocardial stunning from multiple shocks. The patient did not undergo a coronary angiogram hence ischemia was unable to be fully excluded as a precipitating cause behind her PVT; however, she did not have any troponin elevation and there were no ST-T changes on repeat EKGs. Family history was not significant for premature coronary artery disease, arrhythmia, or sudden death. After extensive discussion with family members, it was revealed that the patient had recently started taking an OTC fat burner that contained high amounts of raspberry ketones. The patient was discharged on a life vest and followed up with Cardiology. She underwent an outpatient cardiac electrophysiological study which was unremarkable, and she had a repeat echocardiogram which showed normal cardiac function with normalization of her left ventricular ejection fraction. Due to her uncomplicated post-discharge course life vest was removed.

## Discussion

PVT is a type of arrhythmia characterized by multiple foci in the ventricles resulting in QRS complexes that vary significantly in amplitude, duration, and morphology. Differential diagnosis of PVT includes cardiac ischemia, cardiomyopathies, electrolyte abnormalities, electrophysiologic abnormalities (short or long QT syndromes), and certain medications. A ventricular electrical storm also known as an arrhythmic storm is characterized by ≥ 3 episodes of sustained tachycardia or defibrillations within 24 hours. In the acute setting, management of VT may require immediate cardioversion, defibrillation, or administration of antiarrhythmic drugs. Refractory ventricular electrical storm can be terminated with a transvenous pacemaker. Long-term management includes ablation or implantable cardioverter defibrillator (ICD) implantation among whom a completely reversible cause is not identified.

In our patient with a lack of major cardiac risk factors, the run of VT was possibly induced by a combination of the stress of infection and OTC dietary supplements containing high amounts of raspberry ketones. OTC dietary supplements often cause ingredients that can precipitate arrhythmias and sudden cardiac death [1,2]. Dietary supplements for weight loss are becoming more and more popular due to the increasing prevalence of obesity. These supplements are not tightly regulated by the US FDA and oftentimes lack vigorous quality control and safety standards. In the past, many weight loss supplements were withdrawn from the market due to their safety concerns and adverse effects related to cardiovascular complications, risk of suicide, and depression. Fat burners are a type of dietary supplement which is thought to increase fat metabolism and energy consumption and hence facilitate weight loss [3]. One such fat burner is raspberry ketone, which became popular for weight loss after it was mentioned on the Dr. Oz television show during the segment called "Raspberry ketone: Miracle fat-burner in a bottle" in February 2012. Raspberry ketones are structurally related to a stimulant compound called synephrine and are thought to possibly activate the β-adrenergic receptors. Animal studies suggest that raspberry ketones enable weight loss through various mechanisms, including norepinephrine-induced lipolysis [4]. At least one case of coronary vasospasm has been associated with raspberry ketone intake [5]. Investigations of raspberry ketone in quantitative structure-activity relationship (QSAR) models indicated potential cardiotoxic effects [6].

To improve safety and limit adverse effects, dietary supplements need to be more closely regulated. All ingredients in the supplement should be listed on the bottle with their exact amount and associated side effects. Public awareness, strict regulation policies by the government, along with visible clearly stated disclaimers from the manufacturers should be encouraged.

## Conclusions

Resistant PVT can potentially be a rare but life-threatening complication of non-FDA-regulated OTC weight loss supplements. Dietary supplements should pass through strict safety standards and regulations before being approved and they should be monitored post-marketing to detect any rare side effects.

## References

[1] Nazeri A, Massumi A, Wilson JM (2009). Arrhythmogenicity of weight-loss supplements marketed on the Internet. Heart Rhythm.

[2] Inayat F, Majeed CN, Ali NS, Hayat M, Vasim I (2018). The risky side of weight-loss dietary supplements: disrupting arrhythmias causing sudden cardiac arrest. BMJ Case Rep.

[3] Jakopin Ž (2019). Risks associated with fat burners: a toxicological perspective. Food Chem Toxicol.

[4] Morimoto C, Satoh Y, Hara M, Inoue S, Tsujita T, Okuda H (2005). Anti-obese action of raspberry ketone. Life Sci.

[5] Khattar A, Beeton I (2020). Coronary vasospasm and raspberry ketones weight-loss supplement: is there a connection?. Anatol J Cardiol.

[6] Bredsdorff L, Wedebye EB, Nikolov NG, Hallas-Møller T, Pilegaard K (2015). Raspberry ketone in food supplements - high intake, few toxicity data - a cause for safety concern?. Regul Toxicol Pharmacol.

